# Barriers to and Facilitators of Pediatric Palliative Care in Mainland China, Hong Kong, and Taiwan: A Document Analysis

**DOI:** 10.3390/children12111520

**Published:** 2025-11-10

**Authors:** Yajing Zhong, Chris Gastmans, Veerle Labarque, Alice Cavolo

**Affiliations:** 1 Centre for Biomedical Ethics and Law, Faculty of Medicine, KU Leuven, 3000 Leuven, Belgium; 2 Centre for Molecular and Vascular Biology, Faculty of Medicine, KU Leuven, 3000 Leuven, Belgium; 3 Department of Pediatric Hemato-Oncology, UZ Leuven, 3000 Leuven, Belgium; 4 Institute of Biomedical Ethics and History of Medicine, Faculty of Medicine, University of Zurich, CH-8006 Zurich, Switzerland

**Keywords:** pediatrics, palliative care, barriers, facilitators, guidelines, mainland China, Hong Kong, Taiwan

## Abstract

Background: Although progress has been made, substantial barriers exist in the development and implementation of pediatric palliative care (PPC) in mainland China, Hong Kong, and Taiwan. Possible explanations include the idea that cultural taboos erect barriers that short-circuit PPC discussions among stakeholders, and secondly, regional guidelines intended to support PPC fail to do so effectively. Here, we aimed to identify and document the scope of barriers to and facilitators of PPC practices in these regions and to explore to what extent and how regional PPC guidelines address these barriers/facilitators. Methods: We identified and compared two kinds of documents: (1) recent journal articles reporting on empirical studies of barriers to/facilitators of PPC practices in mainland China, Hong Kong, or Taiwan and (2) published PPC regional guidelines from the three regions. International and regional databases were searched to identify articles, along with PPC professional organizations in the three regions to identify PPC guidelines. Inductive content analysis was used for data analysis, synthesis, and document comparison. Results: Seventeen relevant articles on PPC barriers/facilitators and 16 documents with PPC guidelines were identified. Barriers/facilitators were reported on three organizational levels: micro, meso, and macro levels. Micro refers to children and parents, meso to medical institutions and healthcare providers (HCPs), and macro to policy/guidelines and networks. Most barriers were addressed by PPC guidelines, and most facilitators were acknowledged and endorsed in the guidelines. For instance, HCPs reported that insufficient PPC-related knowledge among stakeholders was a barrier, while PPC guidelines provided detailed information to address this shortfall. Unaddressed barriers in the guidelines were also uncovered, such as the cultural taboo of discussing death, suggesting that HCPs often struggled to have effective PPC conversations with parents and the child. Conclusions: Our finding that PPC guidelines addressed most barriers/facilitators while HCPs still struggled with implementing PPC indicates that mature PPC guidelines are necessary but not sufficient for PPC practices to improve in these three regions. The wide availability of PPC guidelines and PPC education/training for HCPs and families needs to improve.

## 1. Introduction

Mainland China has the second largest child population (aged 1 to 18 years old) in the world, accounting for 13% of the global child population and 21% of the national population [1]. The under-five mortality rate in mainland China is 6.6 (per 1000 live births) [2]. Taiwan has 3.9 million children, about 16.5% of its total population [3], and the under-five mortality rate is 4.4 (per 1000 live births) [4]. Hong Kong has over 960,000 children, accounting for 13.6% of its total population [5], and the under-five mortality rate is 1.9 (per 1000 live births) [6]. With the large child populations and relatively high child mortality rates, children’s quality of life (QoL) at the end of life (EOL) should be considered a priority in the healthcare system.

Over the past few decades, the survival rate of children with serious illnesses has dramatically increased [7], mainly due to advances in modern medicine. However, many children still suffer from serious disease-related complications [8]. When improvements in survival or QoL are no longer possible, ongoing life-sustaining treatments (LSTs) may not be in the best interest of these children [9]. Thus, in some cases, providing pediatric palliative care (PPC), rather than curative care, is ethically and clinically advisable to improve the QoL of these children and their family [10].

PPC encompasses comprehensive care for both the child with a life-threatening condition and for their family. This includes providing physical relief as well as psychological, social, and spiritual support [10]. Since the 1970s, PPC has progressed in many countries and regions [11]. However, in some regions, like mainland China, Hong Kong, and Taiwan, the development of PPC is relatively new. Although PPC in Taiwan has reached the highest level in Asia, it only started to become widely available after 2016 [12]. In Hong Kong, PPC has been supported substantially since 2011, but it is still underdeveloped and has not yet become a specialty service in public hospitals [13]. In mainland China, PPC has developed slowly since 2010 [14]; yet, it is still in its infancy as a routine practice [15,16,17,18,19,20].

In our previous studies [21,22,23], we uncovered practical barriers in physicians’ attitudes toward implementing PPC. Most prominently, these barriers include lack of supportive PPC guidelines, especially in the Chinese context. Hence, in the present study, we aimed to document the scope of barriers to and facilitators of PPC practices in mainland China, Hong Kong, and Taiwan in relation to guidelines meant to aid PPC practices. We specifically sought to better understand (1) to what extent and how barriers/facilitators are addressed in the PPC guidelines of the three regions and (2) which barriers/facilitators are not addressed in the guidelines. Moreover, although providing extensive recommendations is beyond the scope of this article, our results nevertheless prompted us to make some suggestions on how to better address the barriers that were not mentioned in the guidelines or were sparsely discussed.

## 2. Materials and Methods

### 2.1. Data Collection

We were interested in identifying empirical studies on barriers to/facilitators of PPC in mainland China, Hong Kong, or Taiwan and PPC guidelines that address those barriers/facilitators. We defined “guideline” as an institutionally authorized document (e.g., procedures, protocols) that provides physicians and other caregivers with a decision-making process or phased plan for addressing clinical–ethical problems in children with life-limiting conditions [24]. Our search strategy and inclusion criteria are presented in Box 1. Firstly, we searched six databases (Medline^®^, Google Scholar, China National Knowledge Infrastructure, Central Library Taiwan Periodical Literature, Taiwan Scholar Journal Database, and HKInChip) with the aim of identifying all empirical studies conducted in mainland China, Hong Kong, or Taiwan that investigated the barriers/facilitators in the three regions, with perspectives from healthcare providers (HCPs) and/or the family (parents and children). This process identified a good number of empirical studies on PPC barriers/facilitators. Secondly, we searched the same six databases for PPC-related guidelines in the three regions. However, the guideline search yielded no relevant results in three databases (Central Library Taiwan Periodical Literature, Taiwan Scholar Journal Database, and HKInChip). To ensure a comprehensive guideline retrieval, we therefore extended the search to five additional databases: Embase^®^, Web of Science™, Cinahl^®^, CQVIP, and Wanfang Data. Additionally, to identify any other candidate or overlooked PPC guidelines originating in mainland China, we emailed the regional associations/organizations that dealt with palliative care to aid our search. These relevant organizations were from the mailing list we used in our earlier empirical-survey study [23]. Finally, we searched the websites of government, health ministry, and PPC-related associations/organizations in the three regions for relevant PPC guidelines.

### 2.2. Data Extraction, Analysis, and Synthesis

We used the methods of inductive content analysis to extract, analyze, and synthesize data [25]. The process was conducted collaboratively by three authors. YZ performed the initial steps, which were then reviewed and discussed with CG and AC through team meetings. Any disagreements were settled by discussions until consensus was reached. Four steps were involved: (1) We first repeatedly read the articles and guidelines to familiarize ourselves with the content. YZ, who is fluent in English and is a native Chinese speaker, translated any documents written in Chinese into English, the language in which the data analysis was conducted. In this case, CG and AC could participate in the data analysis. The translation documents were used throughout the whole data extraction, analysis, and synthesis process. (2) We then summarized the relevant text into narrative form to identify the main themes of each study and PPC guideline(s). (3) Next, we summarized the main themes, categorizing them into two general conceptual schemes: one summarizing the main themes identified from the empirical studies on barriers/facilitators and another one summarizing the main themes identified from the PPC guidelines. (4) Finally, we constructed a table that listed all the barriers/facilitators in one column and the content of the PPC guidelines that addressed these barriers/facilitators in the other column. This allowed us to compare the content of the literature on barriers/facilitators to the content of the guidelines. This table also served as the basis to report the comparative analysis results (Supplementary File S1).

## 3. Results

### 3.1. Characteristics of the Included Articles

Thirty-three publications met our inclusion criteria. We identified 17 journal articles that reported on empirical studies on PPC-related barriers/facilitators [13,17,20,23,26,27,28,29,30,31,32,33,34,35,36,37,38] and 16 PPC-related guidelines [12,39,40,41,42,43,44,45,46,47,48,49,50,51,52,53].

All 17 articles of empirical studies were published after 2008, and 4 were published in 2023 [33,34,35,36]. Ten studies were conducted in mainland China [17,20,23,29,32,33,34,35,36,37]. Nine studies used qualitative methods [17,26,27,28,33,34,35,37,38]. Eleven articles reported on perspectives of HCPs [13,20,23,26,30,31,32,34,35,36,37], three reported on perspectives of parents [2,7,17,28], and the last three reported on perspectives of multiple stakeholders collectively (e.g., HCPs, parents, and children) [29,33,38]. Characteristics of the 17 empirical studies are summarized in Table 1.

Of the sixteen guidelines, six originated from Taiwan [12,49,50,51,52,53] and five each from mainland China [39,40,41,42,43] and Hong Kong [44,45,46,47,48]. Most of the guidelines focused on palliative care in general; we only extracted content dealing with PPC practices (n = 9) [12,39,44,45,46,47,48,49,52]. All guidelines were published in Chinese; six of them also had an English version [44,45,46,47,48,49]. Characteristics of the 16 guidelines are summarized in Table 2.

### 3.2. Main Findings

Our document analysis identified barriers/facilitators to PPC practices that could be characterized on three levels: micro, meso, and macro. Micro refers to the individual level, in our case, barriers /facilitators related to children or parents. Meso refers to the group level, in our case, barriers /facilitators related to medical institutions or HCPs. Macro refers to the community or societal level, in our case, barriers /facilitators related to PPC policy/guidelines and networks.

#### 3.2.1. Micro Level: PPC Barriers/Facilitators Related to Children and Parents

Among the micro-level barriers/facilitators, we identified two themes: child-related barriers/facilitators and solutions and parent-related barriers/facilitators and solutions. “Solutions” refer to proposals in the guidelines for overcoming barriers or enhancing implementation of facilitators.

##### Child-Related

HCPs and parents both reported every child-related barrier/facilitator. All exemplars of these child-related barrier/facilitators were reflected in the PPC guidelines.

*Communication barriers of children*

Four empirical studies found that both HCPs and parents say that children have difficulty in effectively communicating about their condition(s), distress, and needs [20,33,34,35]. This barrier was attributed to their young age and consequent lack of language development and lack of PPC-related knowledge [20]. On the other hand, at older ages, as children have more robust language development [33,35] and ability to express their needs [33], these enhanced abilities were considered to be facilitators of PPC practices.

Our content analysis identified six solutions described in the PPC guidelines that addressed the communication barrier. The first solution was to establish a PPC-needs assessment and identification system [12,40,52]. For a child who cannot express their needs clearly, a second solution was to address barriers related to medical support; specifically, clearly implement a way to comprehensively assess the child’s medical conditions [40].

A third solution was to respect the autonomy of the child [12,41,43,44,45,46,47,53]. HCPs should respect the child’s rights to plainly receive important medical information, and they should communicate openly with the child [43,44,46,53]. To do so, HCPs also need to consider the child’s wishes, needs, age, cognitive capacity, and family and social culture before disclosing information [12,39,40,41,43,44,46,47,53]. For instance, maturity and competence are important factors. Along these lines, HCPs are advised to involve older children in PPC decision-making and to seriously consider their requests [45,53]. This does not imply that younger children should be completely excluded from the relevant discussions. HCPs are also advised to adapt their communication methods according to the maturity of the child so that the information are easily understandable [43,44,46,53]. This includes using non-verbal types of communications such as communicating through painting [53]. Children should also be allowed to choose with whom they prefer to talk [53].

A fourth solution to overcome communication barriers of young children was that HCPs should help parents discuss the concept of death with the child. To do so, HCPs were advised to choose an appropriate time and place, use understandable language [43,53], and leave time for the child and parents to talk, reflect, and ask questions [46,53]. A fifth solution was to provide psychological support for the child so that they could express their needs [53]. A final solution was to provide spiritual support for the child. In this regard, HCPs were advised to respect the child’s culture, religion, and values [40,43].

*Children’s poor medical condition*

Two studies identified children’s poor medical conditions as a barrier to PPC practices [34,35]. In some cases, a child’s medical condition deteriorated acutely. However, the parents would not accept the reality that their child was dying. This perspective tended to cause parents to want to continue LST, which, in effect, hindered PPC practices [34]. Conversely, two other studies identified a child’s stable medical condition as a facilitator of PPC. This allowed the child to express their needs better, according to both HCPs’ and parents’ perspectives [27,33].

One solution proposed was to assess the child’s condition regularly so that any changes in the condition and preferences of the child and parents were fully considered, new needs and problems were addressed, and sufficient information was obtained to aid decision-making [43,47,53].

*Children’s involvement in decision-making*

Five studies identified the child’s involvement in PPC decision-making as a facilitator of PPC practices [27,28,31,33,35]. This point was reflected in the guidelines, which suggested always involving the child in the decision-making [12,39,40,41,43,44,46,47,50,51,53]. More specifically, physicians should take the lead in facilitating discussions on medical decision-making [46], but these decisions should be made only after reaching a consensus with the child and parents [53].

##### Parents

All parent-related barriers/facilitators we identified were reported by both HCPs and parents. Most of these were reflected in the guidelines, except for two barriers and one facilitator. The two barriers were (1) parents had unrealistic hope in good outcomes that might lead to conflicts with HCPs and (2) limited communications between parents and the child.

*Parents’ lack of PPC-related knowledge*

Five studies found that both HCPs and parents identified the lack of parents’ PPC-related knowledge as a barrier to PPC practices [20,29,34,35,38]. Parents had difficulty accessing PPC-related information [34,35]. They often wrongly assumed that PPC was only relevant for cancer patients or older adults with untreatable illnesses [38].

Eleven PPC-related guidelines emphasized the importance of involving parents in the PPC decision-making and respecting their autonomy [12,39,40,41,43,44,46,47,50,51,53]. To achieve that, HCPs should provide plain-language information about the therapeutic options available to the child [41,43,44,45,46,49,53] and ensure that parents have access to adequate PPC-related information [53].

*Parents’ distress*

In one study, HCPs identified parents’ distress about their child (e.g., feeling lonely, fearful, panic, desperate) as a barrier to PPC practices [34]. HCPs and parents both stated that psychological and spiritual support for parents were PPC facilitators [17,34].

Two PPC guidelines acknowledged that HCPs should help parents to cope with their child’s life-threatening conditions [43,53]. Six guidelines proposed three solutions that would help deal with parents’ distress and effectively help them [12,40,43,46,52,53]. First, the aim should be to provide psychological support [40,46,53] and to provide emotional support and grief counseling [40,53]. Second, they should provide parents with spiritual support appropriate for their culture, religion, and values [43]. Third, they should help parents find solutions when there is a lack of social support [12,40,43,46,52,53]. Specifically, HCPs should consider the social and financial burden of the family [39], provide short-term accommodation for families traveling from remote areas [12,52,53], and provide a free funeral in these cases [12,52,53].

*Community of families experiencing similar conditions*

Another parent-related facilitator was connecting with other families undergoing similar experiences. One study found that putting the family in contact with other similar families could facilitate PPC practices [37]. This helped the child and parents realize that others were having the same unpleasant experiences, which, in effect, created a community on which they could rely, commiserate, and feel needed, all bolstering mental health [54].

Three guidelines recommended the solution of connecting similar families so that they could freely communicate, share experiences, gain emotional support, and help each other in general [12,52,53].

*Other barriers/facilitators*

We identified two additional parent-related barriers to PPC practices at the micro level. Firstly, parents might have an unrealistic hope for a good outcome for their child, which could lead to conflicts with HCPs [28]. On the other hand, parental acceptance of their child’s imminent death could be a facilitator [28]. The second barrier was limited communications between parents and the child [17,29,33,35,37]. Due to poor communication, parents underestimated their child’s suffering [29,33,35,37]. On the other hand, the shared culture and background between the parents and their child, as well as the family’s support and respect for the child, could be a facilitator [33,35,37], as these went a long way to tempering family disputes [33]. Moreover, assistance in helping parents curate the legacy of their child has been reported to be a facilitator [37]. Unfortunately, we have uncovered no guidelines addressing this last group of barriers/facilitators of PPC implementation.

#### 3.2.2. Meso Level: PPC Barriers/Facilitators Related to Health Institutions and HCPs

Among the meso-level barriers/facilitators, our document content analysis identified two themes: health institution-related barriers/facilitators and solutions and HCP-related barriers/facilitators and solutions.

##### Health Institutions

At this level, most of the barriers/facilitators related to health institutions were reported by HCPs; some were also reported by the family. Except for the lack of PPC financial support in medical institutions, all barriers/facilitators were reflected in the PPC guidelines.

*Lack of PPC-dedicated hospital units*

Nine studies found that both HCPs and parents stated that very few medical institutions supported PPC practices [13,20,23,30,31,32,34,35,38] due to limited PPC-related resources [13,20,23,30,34,35]. Facilitators included PPC options provided by some medical institutions [35], home-like PPC hospital wards [33], and supportive resources (e.g., book club) [37].

Six solutions were proposed in the PPC guidelines for this meso-level barrier/facilitator [12,40,41,43,45,46,47,50,51,52,53]. First, some guidelines suggested that PPC should be incorporated into the medical institutions [12,45,47,52]. The guidelines outlined five ways on how to achieve that solution: (1) to establish PPC teams in medical institutions [12,52]; (2) to include PPC practices as a metric for care quality evaluation [12,52]; (3) to provide a detailed workforce plan for instituting PPC [47]; (4) to routinely provide ethics consultation [45]; and (5) to provide legal advice [45].

A second solution in the PPC guidelines for this meso-level barrier/facilitator was to fully equip institutions with appropriate instruments for carrying out PPC [51]. A third solution was to provide environmental support for PPC [40,47,51]. Environmental support includes creating a comfortable, quiet, family-oriented and human-centered environment [40,47] that incorporates complementary therapies (e.g., play therapy, art therapy) [51]; placing the dying child in a single room or independent space, where the family and child can have a private PPC experience [40]; and providing a flexible visitation arrangement that opens up the visitation hours for the family, perhaps even allowing overnight stays [40,47].

A fourth solution in the PPC guidelines for this meso-level barrier/facilitator was to ensure adequate care staffing [43,47]. Specifically, this meant guaranteeing that at least one contact person from the PPC team would be available 24/7 [43]. A fifth solution was to establish a PPC network in which multidisciplinary PPC teams in large tertiary hospitals would function as the specialized center, and smaller hospitals would provide secondary care in the pediatric network [12,40,41,43,46,47,50,51,52,53]. The sixth solution in the PPC guidelines for this meso-level barrier/facilitator was to provide appropriate PPC pharmaceutical resources to the medical institutions providing PPC [51].

##### Healthcare Providers (HCPs)

Most of the barriers/facilitators at the meso level that were related to HCPs were reported by HCPs themselves, but some were also reported by parents. Most of the issues were addressed in the PPC guidelines. Moreover, our analysis found that most included documents clearly stated the main principles that guide PPC practices and EOL decision-making.

*Lack of knowledge about PPC fundamental principles*

Lack of PPC knowledge was also identified at the meso level. Three studies reported HCPs’ lack of knowledge about PPC fundamental principles as a barrier [26,30,31]. For instance, medical/nursing students misunderstood PPC to be prolonging life or misunderstood it to be synonymous with EOL care [31].

Nine guidelines proposed respecting PPC ethical principles to be a solution to this knowledge barrier [12,39,41,42,45,46,48,52,53]. In particular, it was found that HCPs should respect the ethical principle of beneficence and non-maleficence [39,41,42,45,46,48,53]. They should assess the benefits and burdens of LST and decide on a case-by-case basis whether to continue or withhold/withdraw LST [39,41,42,45,46,48,53]. Additionally, HCPs should uphold the principle of justice and ensure that every child has the opportunity to receive PPC [12,46,52].

*Lack of PPC-related knowledge and skills*

In 11 studies, both HCPs and parents identified HCPs’ lack of PPC-related knowledge to be a barrier to PPC practices [13,20,23,26,30,31,32,34,35,36]. This included HCPs’ limited knowledge of caring for a dying child [13,29,30,31], not knowing their duties in PPC practices [31], and failing to understand how to do PPC referral [31].

Six studies identified the lack of HCPs’ PPC-related skills as another barrier [13,23,25,32,34,35]. For instance, deficient communication skills [13,26,32,35] hindered conversations between HCPs and the child [17,35]. On the other hand, some studies reported that good communication between HCPs and the family was a facilitator [17,27,35,37], because good communication helped HCPs to better understand the child’s opinions and helped them to be empathetic toward the parents and the child [17,27,37]. Six solutions were proposed in the PPC guidelines to enhance HCPs’ PPC-related knowledge and skills. Firstly, PPC-related training at both basic and advanced levels needs to be implemented [40,41,47,50,51,53]. This basic training requires HCPs to learn to effectively communicate with the child and parents, to understand the child’s condition and needs, and to raise their general knowledge and awareness of PPC [47]. Advanced training needs to be systematic and comprehensive [40,51,53].

Secondly, PPC guidelines and education/training manuals need to be developed so that HCPs can quickly and consistently consult on PPC issues [51]. Thirdly, training in symptom assessment and management needs to be completed [12,39,40,41,42,43,52,53]. For example, HCPs need to know how to assess pain [12,40,42,43,52,53], nutritional status [41], and carryout medication management [39,53]. Fourthly, PPC standard operating procedures need to be developed. This includes four phases: early-stage PPC initiation, continuous care, decision to withhold/withdraw LST, and bereavement care and support [12,39,40,41,42,43,45,46,47,50,51,52,53]. Fifthly, physicians should lead EOL decision-making, and nurses should coordinate interdisciplinary communications [47,53]. Sixthly, a PPC referral mechanism needs to be established [50].

*Lack of professional and emotional support for healthcare providers (HCPs)*

Lack of professional and emotional support was also identified as a barrier at the meso level. Six studies identified the lack of this kind of support for HCPs being a barrier to PPC [13,20,30,31,32,34].

Only one guideline recommended to provide team support to HCPs [40]. To do so, four suggestions can be followed: (1) assess HCPs’ distress level; (2) empathize with HCPs’ fatigue; (3) provide psychological counseling and psychological support; and (4) provide educational tools, including death education and emotion management training.

*Other barriers*

Finally, our document analysis uncovered three additional barriers to PPC practices at the meso level. They were identified but were left unaddressed in the PPC guidelines. The first barrier was HCPs’ lack of PPC-related experiences [23,31,35]. The second was HCPs’ negative attitudes toward PPC [13,23,26,30,31]. Some HCPs were unusually distressed about caring for a dying child [13,26,31]. Lastly, HCPs’ underestimation of the child’s psychological distress was a barrier to carrying out PPC [33,37].

#### 3.2.3. Macro Level: PPC Barriers/Facilitators Related to Policy/Guidelines and Networks

Among the macro-level barriers/facilitators to PPC, our analysis found two themes: policy/guideline-related barriers/facilitators and solutions and network-related barriers/facilitators and solutions. At this level, all barriers were reported by HCPs but only two were addressed in the guidelines.

##### Lack of Supportive PPC-Related Policies/Guidelines

Three studies identified the lack of supportive PPC-related policy/guidelines as a major barrier to PPC practices [20,23,34]. Five documents proposed solutions for developing PPC-related policies/guidelines to address this barrier [12,47,50,51,52]. For instance, one solution was to broadly implement laws and regulations related to the child’s general rights [51]. Other solutions were to establish frameworks to stimulate ethical discussions, to improve communication skills, and to apply PPC to clinical practices [51].

##### Lack of PPC Networks

Two studies recognized that a macro-level barrier to PPC was the lack of PPC-related networks of professionals [20,34]. Six PPC guidelines proposed establishing a PPC network [12,43,47,51,52,53]. To do so, five suggestions would aid this goal. Firstly, designated hospitals should serve as the major providers of PPC [12,47,51,52,53]. Secondly, a community-based network should be created for providing PPC from hospital to community [47,51]. Thirdly, as many families prefer having PPC at home, PPC homecare services should be developed [12,47,52,53]. Fourthly, the network should contain provisions that enhance PPC support for children who still want to go to school [47]. Fifthly, the network should have the capacity to provide long-term care [43]. Instituting these five suggestions would further the accomplishment of improving PPC networks and delivery of PPC.

#### Other Barriers/Facilitators at the Macro Level

Three other barriers to PPC were also identified at the macro level. The first barrier is the uneven geographic availability of PPC-related resources [20]. Most of the PPC teams in the three regions we studied are geographically situated in more economically developed regions and are affiliated with tertiary-level hospitals [20]. Thus, some places can more easily carry out PPC, whereas other places may not even be able to obtain the PPC training required.

The second barrier at this level was the lack of insurance coverage for PPC [20]. The third barrier to PPC was the widespread traditional cultural taboo of discussing death in these three regions [34,35,37]. An unwillingness to discuss death obviously hinders the frank discussions needed to initiate PPC. Indeed, HCPs found it difficult to practice PPC, as parents avoided discussing death with their child. In this case, HCPs focused more on continuing LST beyond when PPC options should be discussed. On the other hand, more recently, a supportive social culture was considered to be a facilitator to PPC [33]. For instance, a culture of respecting children’s rights, autonomy, and privacy represents a supportive facilitator [35]. Education about death and how to promote dignity was also considered to be facilitating [35]. Unfortunately, we have not yet uncovered PPC-related guidelines that address these barriers or refer to these three macro-level facilitators.

## 4. Discussion

Significant barriers still exist to the routine use of PPC in mainland China, Hong Kong, and Taiwan, despite a great need. Our document analysis identified barriers/facilitators on individual, institutional, and societal levels, which can be organized into a micro, meso, and macro hierarchy. Our discovery of some mismatches between barriers/facilitators and PPC guidelines that purported to address these allow us to offer suggestions on how to address shortfalls in PPC implementation. Several findings especially stand out, for instance, the lack of PPC-related knowledge of both HCPs and family. Although interpersonal conflicts are a major barrier to PPC [17,28,35], none of the guidelines we analyzed gave detailed suggestions about resolving conflicts between HCPs and parents, or between parents and the child. We also found some unaddressed barriers in PPC guidelines (e.g., HCPs lack PPC-related clinical experiences, and there is an uneven availability of PPC-related resources within the three regions). These will be discussed in turn.

### 4.1. Stakeholders’ Lack of PPC-Related Knowledge

One of the main barriers identified through our analysis of empirical studies was the lack of PPC-related knowledge of all stakeholders. This implies that the PPC-related education/training should be provided not only to HCPs, but also to the family. Hence, the supportive guidelines should explicitly acknowledge the importance of PPC training/education for the family and that specific PPC training should be developed for and provided to families, not just HCPs.

PPC-related knowledge is the key for improving HCPs’ confidence in initiating PPC discussions with families and providing PPC. However, other studies have noted that many HCPs have poor PPC-related knowledge [20,36]. For instance, in Cai et al., most pediatric oncologists reported having limited understanding about PPC, although they desired PPC education, and only 23% reported they had actually received PPC-related training or had taken courses on PPC [20]. The lack of HCPs’ PPC-related knowledge is correlated with the lack of PPC training/courses for HCPs, as few medical schools in mainland China provide PPC courses [55]. Therefore, in line with the Royal College of Pediatrics and Child Health in the UK and the American Academy of Pediatrics, PPC-related training/courses should be provided both at the university level and as continuing post-graduate education [56,57]. Their easy accessibility is also necessary to help HCPs initiate PPC discussions and facilitate establishing PPC teams.

PPC education for the family is also necessary for PPC improvement. However, achieving this goal could pose unique challenges in mainland China, Hong Kong, and Taiwan. The lack of death education and deeply entrenched spiritual beliefs in the traditional Chinese culture (e.g., Taoism, Confucianism, and Buddhism) about discussing death hinders HCPs from broaching the topic of death with the child or their parents [58,59]. At the macro level, PPC is not widely popular in mainland China, Hong Kong, or Taiwan. To move the use of PPC beyond the developmental stage and to improve implementation of PPC, the first step is to educate the public about death and PPC as a caring practice [60]. For example, educating the public about medical and ethical aspects of death and dignity and creating a more positive social environment for PPC practices could make PPC more acceptable to many living in traditional Chinese societies [35,61]. The government in mainland China proposed using different social media apps to educate people about hospice and dying with dignity as a step in this direction [61]. At the meso level, primary and secondary schools have been encouraged to incorporate education about death into the curriculum for students [61,62]. At the micro level, HCPs could aim to better inform parents that their child is dying through multiple, personalized ways [60]. HCPs could also help the child face EOL to better understand the concept of death by using videos, audios, and peer education. For example, encouraging children with similar EOL conditions to share their experiences and what they know about death and dying with each other might improve their QoL [62].

### 4.2. Lack of Supportive PPC-Related Guidelines

Three studies from mainland China identified the lack of supportive PPC-related guidelines as a prominent barrier to PPC practices [20,23,34]. However, our analysis in the present study uncovered a good number of adequately supportive guidelines originating from the three regions. We hypothesize that the basis for the barrier is not from the lack of guidelines per se but instead from these guidelines not being equally available across all geographical regions. It is also possible that HCPs were unaware of the existence of these guidelines.

In mainland China, PPC-related resources are limited [15]. For instance, there is only one professional PPC association: the PPC subspecialty group of the Pediatrics Society of the Chinese Medical Association [20]. Additionally, PPC-related resources are unevenly distributed over the large landmass of China [63]. There are five specific aspects that pose challenges to PPC practices in mainland China [15]. First, PPC is mainly practiced in economically more developed cities, such as Beijing, Shanghai, Shenzhen, and Changsha [18,20,64,65]. Second, in rural areas that have begun to implement PPC, PPC-related resources are less developed compared to those in urban areas [63]. Third, PPC is mainly provided in oncology hospitals and departments [15,18], leaving out other areas of medicine that have legitimate PPC needs. Fourth, to the best of our knowledge, all but one PPC team at the time of our analysis were affiliated with tertiary-level hospitals [20]. Fifth, healthcare institutions with modern and well-equipped facilities were more likely to practice PPC [36]. In this latter situation, there are secondary effects of this shortfall. The limited and unevenly distributed PPC-related resources across mainland China could lead to the inadequate acquisition of PPC knowledge among stakeholders, regardless of the existence of PPC-supportive guidelines. Therefore, the government would do well to increase investment in improving PPC-related resources across the whole territory [61].

Another issue related to a lack of supportive PPC-related guidelines in general means that most PPC-related guidelines were formulated only in geographical regions that had more PPC-related resources. This might explain why many HCPs considered the lack of supportive guidelines to be a barrier to PPC practices. This situation also shows the importance of not only developing good guidelines but also of ensuring that the guidelines are widely available and that HCPs are well-acquainted with them. PPC or palliative care organizations can greatly help in circulating guidelines throughout all regions. For example, they could freely share the guidelines with hospitals, post them on their organization websites and on social media, or share them publicly at PPC-related and other conferences. Practices of the European Association for Palliative Care (EAPC) could be taken as an example [66]. The EAPC is a membership organization that supports palliative care development throughout Europe and beyond; it aims to establish strong networks that closely collaborate with its members, research partners, and other European and international agencies [66]. The EAPC is a centralized organization that covers a large area, which is one of the reasons why it is so effective in spreading and improving PPC. Hence, something similar could also be effective in mainland China, Hong Kong, and Taiwan.

### 4.3. Interpersonal Conflicts in PPC Practice

In the present study, no PPC guidelines from mainland China, Hong Kong, or Taiwan gave specific suggestions on how to deal with parents’ refusal to discuss death and PPC with their children, or how to approach and resolve conflicts between HCPs and parents.

Although most of the included PPC guidelines from the three regions recommended involving the child [12,39,40,41,43,44,45,46,47,50,51,53] and parents [12,39,40,41,43,44,46,47,50,51,53] in the PPC decision-making process, as well as respecting the autonomy of the child [12,39,40,41,43,44,45,46,47,53] and parents [41,43,44,45,46,49,53], they failed to address how to overcome a poor communication barrier between parents and the child; this, of course, hindered PPC practices [17,35]. The guidelines just stated vaguely that this communication channel is important. Parents struggled to discuss death or PPC with their child [17,35], as they hoped their child would be cured [17], wanted to lower their child’s emotional distress [67], and believed that their child has limited understanding of death and PPC anyway [17].

The lack of communication about death and PPC between parents and their suffering child can be better understood by considering how traditional Chinese culture “thinks” about discussing the topic of death; it is considered a taboo. This situation was an unaddressed barrier in the guidelines. Traditional Chinese philosophies (e.g., Taoism, Confucianism, and Buddhism) discourage talking about death because it is considered “unfortunate” or “unlucky” [58,59,68,69,70]. Moreover, parents assume that HCPs will try all possibilities to prolong a child’s life [71,72]. Taken together, these serve as substantial sociocultural barriers to PPC practices in the Chinese context, causing parents to avoid discussing death with their child.

Theoretically, trained HCPs should help parents discuss death with their child, and some of the included guidelines provided valuable advice on how to achieve that. Two guidelines from mainland China and Taiwan recommended that HCPs impart information about death and PPC using plain, understandable language [43,53]. In line with the American Academy of Pediatrics recommendations [73], our analysis suggests that respectful, truthful, and thorough communication between parents and their child concerning death and PPC could improve PPC practices across different care environments and throughout the course of the child’s disease as it evolves. Specifically, applying care-planning tools like the Voicing My Choices^TM^ tools could enhance the conversations [74]. Additionally, involving spiritual counselors trained in PPC could also be helpful [57,75].

Another reason why parents refuse to discuss PPC with their child (and with HCPs) is that parents hold out unrealistic hope for good outcomes [28]. This kind of mindset not only stifles any kind of discussion, but the unrealistic expectations of parents could actually lead to real discord between HCPs and parents [28]. Guidelines did not address this barrier. This barrier can take several forms. For example, HCPs and parents might disagree about whether and how much information should be disclosed to the child, especially when the prognosis is poor [76]. Interpersonal conflicts between HCPs and parents could erode trust and respect for each other, disrupt the continuity and coordination of care, and blur the decision-making regarding the child [77,78,79,80]. These kinds of conflicts are not unique to PPC, or in Chinese cultures; they are prevalent in neonatal and pediatric EOL care in general. For instance, a systematic review of the literature on shared decision-making in adolescent healthcare found that disagreements between HCPs and parents damaged their mutual trust [76]. Moreover, Cavolo et al. identified doctor–parent disagreements as the main ethical challenge faced by neonatologists when preterm infants are born in the “gray zone” [81]. Although the literature agrees that going to court should be the last resort for resolving HCP–parent conflicts, there is no clear consensus on how to diffuse disagreements before they blow up and the only course of resolve them is to take a legal one in family court [81]. For example, Cavolo et al. found that using ethical frameworks to analyze a case and discuss this with parents could help solve disagreements between HCPs and parents. However, they also found this method to be time-consuming and, therefore, often not actionable [82].

Recently, the Nuffield Council on Bioethics and the Royal College of Pediatrics and Child Health from the UK made suggestions for resolving disagreements between HCPs and parents based on the severity of the conflict. This focus on severity, we believe, is a first step in the right direction [57,83]. For minor disagreements, aiming to communicate in a timely manner could be a key strategy for both parties, and involving psychologists could also help. For moderate disagreements, intervening with third-party opinions of experts might be needed. For high-conflict disputes, legal intervention is often required [83]. Beyond strategies based on severity, however, much could be gained if PPC guidelines gave specific advice on how to communicate in case of disagreements and then provided proper communication training to all stakeholders.

### 4.4. Strengths and Limitations

Although the present study was not a systematic review, we rigorously and systematically searched, collected, and analyzed relevant empirical PPC studies and PPC-related guidelines. To the best of our knowledge, this is the first document analysis that assessed whether and to what degree PPC guidelines aligned with barriers/facilitators of PPC in these three regions. Second, in addition to articles and guidelines published in English, we were able to identify and include articles and guidelines available only in the Chinese language. This is a seldom achieved analysis, which helped us to avoid overlooking relevant documents and provided additional insights. Third, we included articles reporting on studies that described barriers to/facilitators of PPC as reported by HCPs and the family, which allowed us to gain a comprehensive picture of the situation of PPC practices in these three regions.

There were also some limitations. First, the support of a certified Chinese–English translator was not available, meaning that some cultural nuances might have been lost during the translation of Chinese-language documents. However, one author [Y.Z.] is a native Chinese speaker and has eight first-author, peer-reviewed journal articles published in English. This firsthand experience in English as a second language mitigated the lack of having a certified translator. Second, only one eligible article was on an empirical study that involved children. This means that children’s perspectives on PPC barriers/facilitators contributed little to our analysis and are still largely unknown.

## 5. Conclusions

We identified barriers to/facilitators of PPC practices in mainland China, Hong Kong, and Taiwan on the micro, meso, and macro levels. Most of them were addressed in the PPC-related guidelines included in our analysis. However, to make PPC practices more acceptable in these regions, more attention should be paid to making PPC guidelines more widely known, and more recommendations should be included in the guidelines to specifically address barriers. PPC practices will likely align more closely with the remarkable improvements seen in social, economic, and environmental conditions in these regions if PPC-related education is developed and implemented for all stakeholders, focusing on overcoming cultural taboos, if PPC-related resources are increased and more widely distributed, and if communication training on how to resolve disagreements is implemented.

## Figures and Tables

**Table 1:** Characteristics of included empirical studies on PPC in mainland China, Taiwan, and Hong Kong.

Study	Year Published	Region	Method	Participants	Sample Size	Aims
Chen et al. [26]	2008	Taiwan	Qualitative	HCPs *	16	To explore nurses’ acute experiences with caring for children with terminal illnesses
Liu et al. [27]	2014	Taiwan	Qualitative	Parents	16	To explore parental experiences when making a “do not resuscitate” decision for their child who is or was cared for in a pediatric intensive care unit
Chan et al. [13]	2019	Hong Kong	Quantitative	HCPs	680	To examine perceived challenges (e.g., related to knowledge, skills, self, and work environment) of HCPs in providing PPC
Wang et al. [28]	2019	Taiwan	Qualitative	Parents	10	To describe parents’ experiences when caring for a child with cancer at the end-of-life
Zhou et al. [29]	2019	Mainland China	Quantitative	HCPs; parents	51 (23 HCPs; 28 parents)	To explore the status quo of pain management in children with tumors and to identify barriers to pain management
Cai et al. [17]	2020	Mainland China	Qualitative	Parents	16	To understand nonreligious, theistic parents’ spirituality and to explore how parents discuss death with their terminally ill children
Peng et al. [30]	2020	Taiwan	Quantitative	HCPs	264	To evaluate knowledge and attitudes of pediatric clinicians regarding pain management and to describe the barriers to pain management in pediatric and neonatal settings
Wong et al. [31]	2020	Hong Kong	Quantitative	HCPs	244	To explore knowledge, attitudes, and educational needs of preclinical medical and nursing students
Cai et al. [20]	2021	Mainland China	Quantitative	HCPs	170	To investigate the geographic distribution, team structure, and services of PPC teams in mainland China and the level of understanding and implementation of pediatric oncologists regarding PPC
Yao et al. [32]	2022	Mainland China	Quantitative	HCPs	370	To investigate the status quo of pediatric nurses’ knowledge and attitudes of palliative care in 31 Chinese provinces; to establish a reference for pediatric nurses specialized in palliative care
Cai et al. [33]	2023	Mainland China	Qualitative	HCPs; parents	25 (14 HCPs; 11 parents)	To develop a model of dignity for children receiving PPC based on the Chochinov Dignity Model
Cheng et al. [34]	2023	Mainland China	Qualitative	HCPs	21	To explore the influencing factors of pediatric hospice care practices perceived by HCPs and to provide reference for improving the quality of hospice care service
Lin et al. [35]	2023	Mainland China	Qualitative	HCPs	13	To understand HCPs’ knowledge about and practice experience with dignity-conserving care for children with advanced cancer and to establish a reference for improving the quality of dignity-conserving care
Zhu et al. [36]	2023	Mainland China	Quantitative	HCPs	412	To investigate the knowledge, attitude, and behavior of pediatric HCPs regarding PPC; and to explore factors that influence the implementation and development of PPC
Cheng et al. [37]	2024	Mainland China	Qualitative	HCPs	27	To explore spiritual care in PPC from the perspective of HCPs
Wong et al. [38]	2024	Hong Kong	Qualitative	HCPs; parents; children	65 (15 HCPs; 25 parents; 25 children)	To compare and contrast the perceived care needs of children with life-limiting conditions from the perspectives of the children, parents, and HCPs
Zhong et al. [23]	2024	Mainland China	Quantitative	HCPs	325	To determine pediatricians’ perspectives on PPC and to identify factors that influence their perspectives

* HCPs, healthcare providers.

**Table 2:** Characteristics of included PPC regional guidelines.

Included Document	N (%)
Regions	
Mainland China	5 (31.3)
Hong Kong	5 (31.3)
Taiwan	6 (37.5)
Years published	
2000–2009	1 (6.3)
2010–2019	6 (37.5)
2020–2024	9 (56.3)
Type of document	
Legal provision	1 (6.3)
Strategy policy document	5 (31.3)
Ethics advice	3 (18.8)
Clinical practice guideline	6 (37.5)
Commission report	1 (6.3)
Focus of document	
Palliative care in general ^1^	9 (56.3)
PPC	4 (25.0)
Pediatric cancer treatment	1 (6.3)
Pediatric health and care	1 (6.3)
Sedation and analgesia for children	1 (6.3)
Organization publishing document	
Ministry of Health and Welfare	4 (25.0)
Institutes of Health	2 (12.5)
Hospital Authority	4 (25.0)
The Law Reform Commission	1 (6.3)
Physician association	4 (25.0)
Nurse association	1 (6.3)
Language of document	
Chinese	10 (62.5)
Chinese and English	6 (37.5)
Length of document	
Median complete document	36 pages (min, 3; max, 680)
Median relevant to PPC	3 pages (min, 1; max, 67)

^1^ These pediatric care guidelines have specific sections about PPC or information relevant to PPC. The data we analyzed were only from sections/information relevant to PPC.

## Data Availability

All data generated or analyzed during this study are included in this published article and its Supplementary Information files. Further inquiries can be directed to the corresponding author.

## Supplementary Materials

The following supporting information can be downloaded at https://www.mdpi.com/article/10.3390/children12111520/s1, File S1. Comparisons of the barriers/facilitators and suggestions identified in PPC-related guidelines.

## Abbreviations

EAPC	European Association for Palliative Care
EOL	End-of-life
HCPs	Healthcare providers
LST	Life-sustaining treatment
PPC	Pediatric palliative care
QoL	Quality of life
