# Supplementary material for: Barriers to and Facilitators of Pediatric Palliative Care in Mainland China, Hong Kong, and Taiwan: A Document Analysis
Source: Children (Basel). 2025 Nov 10;12(11):1520. doi: 10.3390/children12111520 (PMC12651054; doi:10.3390/children12111520)
Supplement: Supplementary file 1 [file children-12-01520-s001.zip › children-3936482-supplementary.pdf]

Supplementary File 1. Comparisons of the barriers/facilitators and suggestions identified in PPC-related guidelines.

**Micro level**	
Children	
Children have difficulty in communicating their conditions, symptoms, distress, and needs effectively (Cai et al., 2021; Cai et al., 2023; Cheng et al., 2023; Lin et al., 2023) due to their young age and lack PPC-related knowledge (Cai et al., 2021).	**Establish a PPC needs assessment and identification system** (Mainland 2, Taiwan 4-5).<br><br>**Address issues related to medical support, e.g., providing** appropriate treatment (Mainland 2).<br><br>**HCPs help parents to discuss death with the child.** They let the child choose the HCPs who they prefer to talk to (Taiwan 6) and communicate with the child in understandable ways (Hong Kong 1, 3, Mainland 5, Taiwan 6), e.g., choose appropriate timings and places, use understandable and acceptable words and expressions (Mainland 5, Taiwan 6) or non-verbal communications (i.e., painting) (Taiwan 6), and leave time for the child and parents to consider the information given and ask questions (Hong Kong 3, Taiwan 6).<br><br>**Respect the autonomy of the child** (Hong Kong 1-4, Mainland 3, 5, Taiwan 5-6). **Consider the child's wishes, age, cognitive capacity, and family and social culture** before disclosing information to them (Hong Kong 1, 3, Mainland 1-3, 5, Taiwan 5-6).<br>• **Assess the child's understanding of death** with assessment tools (Mainland 5).<br>• **Respect the child's right to know important medical information and** communicate with them honestly and openly (Hong Kong 1, 3, Mainland 5, Taiwan 6).<br>• **PPC and the care plan should be provided based on the child's needs** (Hong Kong 4, Mainland 5, Taiwan 5-6). **Younger children (<7 years)** might express wishes and preferences verbally and non-verbally, though they cannot make decisions (Mainland 3, 5).<br><br>**Psychological support for the child.** Provide chances for the child to express their needs and help them understand that their life is important to their family (Taiwan 6).<br><br>**Spiritual support for the child.** The PPC team should respect the culture, religion, and values of the child and parents (Mainland 2, 5).

• The child's age[1] (Cai et al., 2023; Lin et al., 2023)<br>• The child's self-awareness to express needs (Cai et al., 2023)	
The child's poor medical condition (Cheng et al., 2023; Lin et al., 2023)<br><br>The child's medical condition (Liu et al., 2014; Cai et al., 2023)	**Regularly revisit the child's medical condition with the child and parents because it changes over time** (Hong Kong 4, Mainland 5, Taiwan 6). Assess the child's medical conditions with assessment tools (Mainland 5).
Involve the child in decision-making (Liu et al., 2014; Wang et al., 2018; Wong et al., 2020; Cai et al., 2023; Lin et al., 2023	**Involvement of the child in decision-making (**Hong Kong 1, 3-4, Mainland 1-3, 5, Taiwan 2-3, 5-6).<br>• **The child** (Mainland 1, 3) **and their family (parents and siblings)** (Hong Kong 4; Taiwan6) **should be involved in, and at the center of, the decision-making** (Hong Kong 1, Mainland 2, 5, Taiwan 6). **PPC and a care plan should be provided based on the child's needs and wishes** (Hong Kong 4, Mainland 2, 5, Taiwan 5-6). **Mature children's** choices and decisions should be taken seriously (Hong Kong 2, Taiwan 6). Older children have the right to their own opinions (Hong Kong 2).<br>• Doctors take the lead in making medical decisions (Hong Kong 3).
Parents	All parent-related barriers were reported by both HCPs and parents. Most of the parent-related barriers were addressed by the policy/guideline documents, except parents' excessive hope in good outcomes and the lack of communication between the parents and the child. No document gave concrete indications on what to do when the parents and the child have different opinions.
Excessive hope in a miracle/good outcome on the part of the parents (Wang et al., 2018)<br><br>Parents accept the death of their child (Wang et al., 2018)	
Lack of PPC-related knowledge of parents (Zhou et al., 2019; Cai et al., 2021; Cheng et al., 2023; Lin et al., 2023; Wong et al.,	Guidelines Hong Kong 1, 3-4, Mainland 1-3, 5, and Taiwan 2-3, 5-6 clearly state the importance of involving parents in the PPC decision-making.<br>• **The child** (Mainland 1, 3) **and parents** (Hong Kong 4) **should be involved in, and at the center of, the**

2023), partially due to a lack of PPC-related information available for parents (Cheng et al., 2023; Lin et al., 2023)	**decision-making** (Hong Kong 1, Mainland 2, 5, Taiwan 6).<br>• Doctors are responsible for **providing the child and family with adequate information about therapeutic options** (Hong Kong 3).<br>• **The autonomy of the parents must be respected** (Hong Kong 1-3, Mainland 3, 5, Taiwan 1, 6); **parents' right** to know important medical information **must be respected** (Hong Kong 1, Mainland 5); parental consent must be obtained (Taiwan 6).<br>• **Conflict mediation**. The PPC team help to reach consensus if there are disagreements between the family and HCPs. Options include family meetings, specific conversations, and legal advice (Hong Kong 2, Mainland 5).
Parents' distress (Cheng et al., 2023)<br><br>Psychological support for parents (Cai et al., 2020)<br><br>Spiritual support (faith, religious beliefs) for parents (Cai et al., 2020; Cheng et al., 2024)	**Psychological support for parents** (Mainland 2, Taiwan 6). Examples include assessing the challenges faced by the family and their ways of and preferences for communication (Mainland 2); providing emotional support and grief counseling (Mainland 2, Taiwan 6); and encouraging parents to discuss the issue with relatives and close friends (Hong Kong 3).<br>**Addressing issues related to social support** (Hong Kong 3, Mainland 2, 5, Taiwan 4-6). Considering the **social and financial burden** on the family (Mainland 2). Examples include collecting information on financial assistance provided by the government and social organizations (Mainland 5, Taiwan 4-6); providing short-term accommodation for families coming from remote regions (Taiwan 4-6); and providing free funerals (Taiwan 4-6).<br><br>**Helping parents to go through the saddest period** (Mainland 5, Taiwan 6). Examples include respecting **the privacy of the parents** and providing them with space and time for resting and releasing emotions (Taiwan 6); **helping parents to accept the child's death** by, e.g., writing a card to say goodbye to the child and taking photos together (Taiwan 6); and a**ssisting the family in preparing the funeral** (Mainland 5).<br><br>**Spiritual support for the child and parents.** The PPC team should respect the cultural and religious values of the child and parents (Mainland 5).
Parents lack communication with the child (Cai et al., 2020; Lin et al., 2023). They underestimate the	

child's suffering (Zhou et al., 2019; Cai et al., 2023; Lin et al., 2023; Cheng et al., 2024), e.g., the child's pain (Zhou et al., 2019; Lin et al., 2023), psychological distress (Cai et al., 2023), and spiritual needs (Cheng et al., 2024). Conflict of dignity perceptions between the parents and the child could lead to dilemmas (Cai et al., 2023)<br><br>Family accompanying and supporting the child (Cai et al., 2023; Cheng et al., 2024)<br>Family culture/atmosphere of respecting the child's dignity (Cai et al., 2023; Lin et al., 2023).<br>Resolving family disputes (Cai et al., 2023).	
Assistance for parents in the legacy of their child (Cheng et al., 2024)	
Community of childred and parents with similar conditions (Cheng et al., 2024)	**Introduce other families in a similar situation to the child and parents** (Taiwan 4-6).
**Meso level**	Most of the meso-level barriers were reported by HCPs; some were also reported by parents. Most barriers were addressed by the policy/guideline documents.
Medical institutions.	Most of the barriers were reported by HCPs. Most of these barriers were addressed by the policy/guideline documents, except the lack of financial support for PPC implementation in medical institutions. Though two documents highlighted the importance of having enough manpower, no document indicated how to improve human resources.
Limited medical institutions or organizations support PPC practice (Chan et al., 2019; Peng et al., 2020; Wong et al., 2020; Cai et al., 2021; Yao et al., 2022; Cheng et al., 2023; Lin et al., 2023; Wong et al., 2023; Zhong et al., 2024)	**Integrate PPC into medical institutions** (Taiwan 4-5). **Establish PPC teams** in medical centers and children's hospitals in each regions and involve PPC as one of the work evaluation indicators (Taiwan 4-5). Additionally, a detailed workforce plan (Hong Kong 4), ethics committee (Hong Kong 2), legal advice (Hong Kong 2), and transportation support for PPC access and between hospitals (Hong Kong 4) also help.

Medical institutions provided PPC options (Lin et al., 2023)	
Lack of PPC-related resources (Chan et al., 2019; Peng et al., 2020; Cai et al., 2021; Cheng et al., 2023; Lin et al., 2023)<br>• Lack of financial support for PPC implementation in medical institutions (Cai et al., 2021)<br><br>• Lack of comfortable, supportive environment (Cai et al., 2021; Cheng et al., 2023; Zhong et al., 2024)<br><br>• Homelike PPC wards (Cai et al., 2023)<br><br>• Inadequate human resources (Chan et al., 2019; Cheng et al., 2023; Zhong et al., 2024)<br><br>• Lack of professional PPC teams (Peng et al., 2020; Cai et al., 2021)	**Provide PPC-related equipment** for the child (Taiwan 3).<br><br>**Institutional support (environment) for the child and parents** (Hong Kong 4, Mainland 2, Taiwan 3).<br>• Create a **comfortable, quiet, family-oriented, and humanized environment** for the critically ill child (Hong Kong 4, Mainland 2) that incorporates complementary therapies, e.g., play therapy or art therapy (Taiwan 3). Place the dying child in a single room or independent space (Mainland 2).<br>• **Establish an open and flexible visitation system for the child** (Hong Kong 4, Mainland 2). Family can visit at flexible hours and stay overnight (Mainland 2; Hong Kong 4).<br><br>**Manpower** (Hong Kong 4, Mainland 5). The PPC team should be available 24 hours and ensure that at least one contact person is available at all times (Mainland 5).<br><br>**Establish multidisciplinary PPC teams** (Hong Kong 3-4, Mainland 2-3, 5, Taiwan 2-6), as the first step (Hong Kong 4).<br>• **Centralized PPC team.** Hong Kong Children's Hospital works as the tertiary referral center, while other hospitals provide secondary and step-down care. Services have to be established under a single governance for coordinated care in the pediatric service network (Hong Kong 4).<br>• **The interdisciplinary team should include all relevant specialties**, both medical (e.g., pediatricians, nurses, psychologists) and non-medical staff (i.e., religious personnel) (Hong Kong 3-4, Mainland 2, 5, Taiwan 3-5). The **original pediatric team** is familiar with the child's condition and **leads the care**, while the PPC team work as the supplement (Taiwan 6).

• Lack of medicines for the child (Cai et al., 2021; Cheng et al., 2023)<br><br>Supportive resources (Cheng et al., 2024)	**Provide PPC resources**, i.e., medication (Taiwan 3).
Healthcare providers	Most of the barriers were reported by HCPs; some of them were also reported by parents. Most barriers were addressed by the policy/guideline documents, except for the lack of communication with the child, the lack of PPC-related experiences of HCPs, HCPs' personal obstacles, and HCPs' underestimation of the child's psychological distress.<br><br>Most guidelines indicated the main principles that guide PPC practice and decision-making. Some only mentioned the principles, especially the clinical ones, but some included in-depth discussions on how to apply them.
Lack of knowledge about fundamental PPC principles (Chen et al., 2008; Peng et al., 2020; Wong et al., 2020)<br><br><br><br><br><br><br><br><br><br><br>Lack of PPC-related knowledge among HCPs (Chen et al., 2008; Chan et al., 2019; Zhou et al., 2019; Peng et al., 2020; Wong et al., 2020; Cai et al., 2021; Yao et al., 2022; Cheng et al., 2023; Lin et al., 2023; Zhu et al., 2023; Zhong et al., 2024)	**Respect the ethical principles** of making EOL decisions.<br>• **Beneficence and non-maleficence** (Hong Kong 2-3, 5, Mainland 1, 3-4, Taiwan 6). Assessment of the benefits and burdens of treatment are key to deciding whether to provide or withdraw treatment (Hong Kong 2-3, 5, Mainland 1, 3-4, Taiwan 6). The child's best interests should be decided on based on their age, the healthcare team's advice, and the child's wishes, opinions, value, and beliefs (Hong Kong 2-3).<br>• **Maintaining justice** (Hong Kong 3, Taiwan 4-5**).** Children should have the chance to receive PPC (Taiwan 4-5).<br><br>**PPC-related courses/training/resources for pediatric HCPs** (Hong Kong 4, Mainland 2, Taiwan 2-3, 6).<br>• **Basic training** is needed for HCPs to be aware of the child's condition and needs and for them to fully communicate with the child and parents (Mainland 3), as well as to raise HCPs' general knowledge and awareness of palliative care (Hong Kong 4).<br>• **Advanced training** (Hong Kong 4, Taiwan 3).<br>▪ Advanced training is needed for **non-palliative care teams working directly** with the child using the shared model (Hong Kong 4).<br>▪ **Systematic and comprehensive training includes training** on professional PPC consultation, symptom assessment and management (i.e., pain), ethical/legal issues, and psychological care (Mainland 2; Taiwan 3, 6).

• Lack of knowledge on how to care for a dying child, including symptom and pain management (Chan et al., 2019; Zhou et al., 2019; Peng et al., 2020; Wong et al., 2020)	▪ **A case collection** of PPC cases should be established for HCP education, training, discussion, and reflective learning as supplementary materials (Taiwan 3).<br>**Guidelines and education/training manuals on PPC** and clinical care should be formulated (Taiwan 3).<br><br>**Symptom assessment and management** (Mainland 1-5, Taiwan 4-6). The documents give detailed information on symptom (Mainland 2, 4-5, Taiwan 4-6), nutrition (Mainland 3), and medication management (Mainland 1, Taiwan 6).<br><br>We have a wealth of guidelines describing clinical management in depth. This is one of the most commonly addressed barriers (Hong Kong 2-4, Mainland 1-5, Taiwan 3-6).<br>**PPC implementation procedure.**<br>• **Phase 1. Early initiation of PPC** (Hong Kong 4, Mainland 3, Taiwan 4-6). PPC can be introduced and started after the diagnosis is made, or it can be carried out simultaneously with therapeutic treatments (Taiwan 5). **ACP should be made as early as possible** (Mainland 3; Taiwan 6).<br>• **Phase 2**. **Continuous care** (Mainland 1-5, Taiwan 4-6). Medications and dosage should be proportional to the pain and condition of the child. It is fundamental to avoid overdosage (Mainland 1, 4).<br>• **Phase 3. Withhold/withdraw LST** (Hong Kong 2-3, Taiwan 6)**.**<br>▪ **Consider treatment futility** for the child (Hong Kong 2-3).<br>▪ **Document and sign DNACPR** (Hong Kong 2, Taiwan 6).<br>▪ **Choose to withhold LST, withdraw LST, time limited trial, limit treatments, or maintain current treatments** according to different conditions (Taiwan 6).<br>• **Phase 4**. **Bereavement care and support** (Mainland 2, 5, Taiwan 4-6).<br>▪ The care team **build up a trusting relationship with parents** (Mainland 5, Taiwan 6).<br>▪ **The expected location of care and death should be discussed** considering the child's wishes, religious beliefs, spirituality, and culture, as well as HCPs' opinions and safety (Taiwan 6).<br>▪ **Good body care** is the key to bereavement care. Examples include cleaning the child's

• Limited knowledge about HCPs' duties in PPC implementation (Wong et al., 2020)<br><br>• Limited knowledge about PPC techniques (Wong et al., 2020)<br><br>• Limited knowledge about PPC referral guidelines (Wong et al., 2020)	body or providing make up for the child (Taiwan 6).<br>▪ **Bereavement care for siblings.** Examples include **introducing** the child's condition to the siblings based on their ages and supporting them in expressing their emotions (Taiwan 6).<br>▪ If necessary, **organ donation arrangements** (Taiwan 6).<br>▪ **Follow-up after death** (Mainland 5, Taiwan 6). Examples include a follow-up phone call (Taiwan 6), encouraging parents to go back to a normal life (Taiwan 6), or introducing them to families with the same/similar experiences (Mainland 5).<br>**Help families seek hospital, community, or professional resources**, etc. (Mainland 5).<br><br>**Roles.** Decision-making is led by doctors and coordinated by nurses (Hong Kong 4, Taiwan 6).<br><br>**Information Technology (IT) support** (Hong Kong 4).<br>• Enhance the IT system to support the workflow, communication, and care coordination along the palliative care process (Hong Kong 4).<br>• Build up a common platform for the documentation of ACP and set up a palliative care database (Hong Kong 4).<br>• Develop tele-care and mobile apps (Hong Kong 4).<br><br>**Establish a PPC referral mechanism** for the child in the terminal stage of cancer and formulate standard PPC operating procedures (Taiwan 2).
Lack of PPC-related skills among HCPs (Chan et al., 2019; Yao et al., 2022; Cheng et al., 2023; Lin et al., 2023; Zhong et al., 2024)<br>• Lack of communication skills (Chen et al., 2008; Chan et al., 2019; Yao et al., 2022; Lin et al., 2023).	**PPC-related courses/training/resources for pediatric HCPs** (Hong Kong 4, Mainland 2, Taiwan 2-3, 6). Refer to basic training and advanced training mentioned above.

Lack of communication with the child (Cai et al., 2020; Lin et al., 2023).<br><br>Communication with the child (Lin et al., 2023), parents (Liu et al., 2014; Cai et al., 2020; Cheng et al., 2024) and the team (Wong et al., 2020).<br>• Respect for the child's opinions (Lin et al., 2023).<br>• Empathy for the parents and the child (Liu et al., 2014; Cai et al., 2020; Cheng et al., 2024).<br>• Agreement on the importance of PPC among HCPs (Wong et al., 2020).<br>• Coordinated interdisciplinary communication among primary nurses (Wong et al., 2020).	**Respect the autonomy of the parents** (Hong Kong 1-3, Mainland 3, 5, Taiwan 1, 6). **Parents are legally and morally entitled to make decisions for their child,** especially for those younger than 7 (Mainland 3, 5, Taiwan 6). Examples include signing the ACP form (Hong Kong 1) or consenting to or withholding LST for the child (Taiwan 1, 6), as long as they are not acting against the child's best interests (Hong Kong 3, Taiwan 1, 6).
Lack of PPC-related experiences among HCPs (Wong et al., 2020; Lin et al., 2023; Zhong et al., 2024).	
HCPs' personal obstacles (Chen et al., 2008; Chan et al., 2019; Peng et al., 2020; Wong et al., 2020; Zhong et al., 2024).<br>• HCPs have negative attitudes toward PPC (Cheng et al., 2023); they find it unpleasant to care for a dying child (Chen et al., 2008;	

2008; Chan et al., 2019; Wong et al., 2020).<br>• Nurses find it difficult to be certain that they did a good job (Chen et al., 2008).<br>• Sense of guilt (Chen et al., 2008).<br>• Fears of adverse effects of opioid analgesics (Peng et al., 2020).<br>• Challenges related to the content of the work and professional roles of HCPs (Chan et al., 2019).<br>• Personal beliefs and losses (Chan et al., 2019).	
HCPs' underestimation of the child's psychological distress (Cai et al., 2023; Cheng et al., 2024).	
Lack of professional and emotional support for HCPs (Chan et al., 2019; Peng et al., 2020; Wong et al., 2020; Cai et al., 2021; Yao et al., 2022; Cheng et al., 2023).	**Team member support** (Mainland 2). Assess team members' distress, empathize with their fatigue, and provide psychological support, including death education, emotion management training, psychological counseling, etc. (Mainland 2).
**Macro level**	On the macro level, all barriers were reported by HCPs. Few barriers were addressed. We found that one of the major barriers was the lack of supportive PPC policies/guidelines. However, we have found supportive guidelines. We assumed that they either are not equally spread across the Chinese territory or that clinicians do not know them.<br><br>Other barriers, e.g., uneven PPC resources, lack of insurance coverage for PPC, and cultural taboos, were not addressed.
Lack of supportive PPC policy/guidelines (Cai et al., 2021; Cheng et al., 2023; Zhong et al., 2024).	**Develop policy/guideline/regulation/framework** (Hong Kong 4, Taiwan 2-5).<br>• Generalize laws and regulations related to the child's rights (Taiwan 3).<br>• Establish a framework for discussing common ethical issues, generalize communication skills, and apply them in clinical situations (Taiwan 3).

Lack of PPC network	**Establish PPC network** (Hong Kong 4, Mainland 5, Taiwan 3-6). With designated hospitals as major institutions providing PPC, local hospitals, primary clinics, health centers, home care institutions, and support schools can be integrated into the PPC system (Hong Kong 4, Taiwan 3-6). Establish a long-term care system and PPC specialized institutions (Mainland 5, Taiwan 3, 6).<br>• **Support local care** to accommodate the preference of the child and family for receiving PPC at home. The PPC team work with the designated care team at the regional level to ensure the continuity of PPC in the pediatric service network (Hong Kong 4).<br>• **Build up a community-based network of PPC** and provide the child with palliative home care from the hospital to the community (Hong Kong 4, Taiwan 3). Relevant non-governmental organizations and patient groups provide complementary support, with their experiences and resources in the community (Hong Kong 4).<br>• **Develop pediatric palliative home care services** (Hong Kong 4, Taiwan 4-6). Many children and parents prefer having PPC at home. Families can access telephone support for consultation and advice when required (Hong Kong 4).<br>• **Enhance palliative care support for special schools and residential schools**; leverage the existing Complexity Community Support Program (Hong Kong 4).<br>• **Long-term care.** During the provision of long-term, continuous care, the PPC team should consider changing the place of treatment and where the child and parents live (Mainland 5).
Uneven PPC resources (Cai et al., 2021)	
Lack of insurance coverage for PPC (Cai et al., 2021)	
Cultural taboo (Cheng et al., 2023; Lin et al., 2023; Cheng et al., 2024)<br><br>***Supportive social culture*** (Cai et al., 2023).	
Death education and dignity maintenance education (Lin et al., 2023)	

[1] Facilitators are highlighted in blue.
